# Transformation from Film to Nanorod via a Sacrifical Layer: Pulsed Laser Deposition of ZnO for Enhancing Photodetector Performance

**DOI:** 10.1038/s41598-017-14592-6

**Published:** 2017-10-27

**Authors:** Sin-Liang Ou, Fei-Peng Yu, Dong-Sing Wuu

**Affiliations:** 1 Department of Materials Science and Engineering, Da-Yeh University, Changhua, 51591 Taiwan, R.O.C.; 2 Department of Materials Science and Engineering, National Chung Hsing University, Taichung, 40227 Taiwan, R.O.C.

## Abstract

A novel fabrication method for single crystalline ZnO nanorods by pulsed laser deposition (PLD) using a chemical-bath-deposited ZnS seed layer is proposed. For the substrate temperature (T_s_) lower than 700 °C, the PLD-ZnO showed a polycrystalline phase and film-type morphology, resulting from the ZnS seed layer with a cubic phase. However, the ZnS film became a sacrifical layer and single crystalline ZnO(002) nanorods can be achieved at T_s_ of 900 °C, where ZnS was decomposed to zinc metals and sulfur fumes. The transformation from ZnO film to nanorod microstructure was demonstrated with the change of ZnS layer into Zn grains. Enhanced performance of the metal-semiconductor-metal photodetectors were fabricated with ZnO/ZnS samples grown at T_s_ of 500, 700, and 900 °C. The responsivities (@1 V and 370 nm) of these three devices were 1.71, 6.35, and 98.67 A/W, while their UV-to-visible discrimination ratios were 7.2, 16.5, and 439.1, respectively. Obviously, a higher light-capturing efficiency was obtained in the 900 °C-grown ZnO/ZnS device owing to its one-dimensional nanostructure with high crystal quality. The results indicate PLD combined with a sacrifical nanostructure is a promising method for obtaining high-quality ZnO nanorods, which paves the way for the fabrication of high performance ZnO-based devices.

## Introduction

ZnO-based materials have attracted much attention owing to their direct wide band gap, high exciton binding energy (60 meV), high mobility, low cost, good thermal stability, nontoxic feature, and abundant resource^1–6^. Recently, the ultraviolet (UV) photodetectors (PDs) fabricated with ZnO-based materials become more and more important because they can be employed for plenty of applications consisting of UV radiation monitoring, automatization, intersatellite communication, flame sensing, missile launching detection, and biochemical analysis^7–15^. For the fabrications of high-performance PDs, the semiconductor material with a good crystal quality is required. In addition, how to improve the responsivity under UV radiation and increase the photocurrent generation are also the main issues for the UV PDs. It is well known that the one-dimensional ZnO nanostructures such as nanorods, nanowires, and nanosheets have a larger surface-to-volume ratio in comparison to bulk and thin-film ZnO materials. Thus, when the PDs are prepared with ZnO nanostructures, the higher photoconductive gains can be obtained since their surfaces possess a lot of trapping sites to adsorb and desorb oxygen molecules^10–19^.

Several growth methods consisting of aqueous solution technique^10,13,15^, chemical vapor deposition^11,12^, vapor–liquid–solid process^14^, hydrothermal technique^16–19^, sputtering^20^, and pulsed laser deposition (PLD)^21–23^ have been proposed to prepare ZnO nanostructures. Usually, ZnO-based nanostructures are prepared via aqueous solution technique, vapor–liquid–solid process, and hydrothermal method. Nevertheless, these ZnO nanostructures grown by the as-mentioned methods are almost polycrystalline with low crystal qualities. Actually, ZnO-based thin films with smooth surfaces and high crystal qualities are easily achieved using conventional physical vapor deposition (PVD) processes such as sputtering and PLD. However, it is difficult to prepare the high-quality ZnO-based nanostructures under both PVD environments. Thus, there are relatively few researches focusing on ZnO-based nanostructures grown by sputtering or PLD. For the preparation of high-quality oxide materials, PLD is indeed an ideal method because the atomic-layer growth will be realized through the adjustment of the laser repetition rate. Besides, the high-energy source particles created by PLD can improve the surface mobility of the ad-atoms^24–26^. As a result, in comparison to magnetron sputtering or reactive RF sputtering, PLD has attracted much attention for growing ZnO-based thin films or nanostructures. As far as we know, a modified PLD technique that is the so-called nanoparticle assisted PLD (NAPLD) has been presented for the preparation of ZnO-based nanostructures^21–23^. Up to now, the PLD-prepared ZnO nanostructures are almost grown by NAPLD without using any catalyst. In NAPLD growth, the nanoparticles are generated in the background gas by laser ablating for the subsequent nanostructure growth^27^. When ZnO nanostructures are grown by NAPLD, a high working pressure (usually higher than 1 Torr) is an essential deposition condition. At such deposition condition, the laser-ablated ZnO species are limited in a small volume. Through the condensation, ZnO nanoparticles are formed onto the substrate. This is different to the thin-film growth by conventional PLD at a very low working pressure (usually at 10^−2^–10^−3^ Torr). Although single crystalline ZnO nanostructures can be prepared by NAPLD, there are two possible drawbacks formed in the NAPLD growth. Firstly, as the nanostructures are deposited at a high working pressure, there could be more defects created at this growth condition, leading to the deterioration of structural, electrical, and optical characteristics of nanostructures. On the other hand, it is well known that the electrical resistivity of ZnO-based materials is mainly governed by the formation of oxygen vacancies. Several NAPLD-ZnO nanostructures are grown at higher O_2_ pressures, which would induce less oxygen vacancies in the nanostructures. Therefore, the electrical properties of NAPLD-ZnO nanostructures could be degraded, limiting their optoelectronic applications.

In our work, a novel fabrication method for the ZnO nanostructure by conventional PLD has been proposed. We prepared the PLD-ZnO layers at various substrate temperatures (T_s_) of 500, 700, and 900 °C on the chemical-bath-deposited (CBD) ZnS seed layers. At present, several studies on ZnO nanostructures combined with ZnS materials were presented, which mainly consisted of ZnO/ZnS core/shell nanostructures^28–30^ and ZnS branches grown on ZnO^31,32^. However, there is almost no research of the ZnO nanostructures prepared on the ZnS seed layers. In this study, when the T_s_ was lower than 700 °C, the PLD-ZnO layers grown on ZnS films exhibited the polycrystalline structure and film-type morphology. However, with increasing the T_s_ to 900 °C, the ZnS film became a sacrifical layer and the single crystalline ZnO nanorods were formed. In other words, the morphological transformation of PLD-ZnO from film to nanorod can be achieved via the formation of the sacrifical layer at a high T_s_ of 900 °C. The interesting transformation mechanism was investigated in detail, as discussed later. Moreover, these PLD-ZnO samples prepared at the T_s_ of 500–900 °C were used to fabricate the metal-semiconductor-metal (MSM) PDs. A superior performance of the PD fabricated with the ZnO nanorods (T_s_: 900 °C) was also demonstrated. Currently, most of the ZnO-based nanostructure-type devices are fabricated by aqueous solution technique, vapor–liquid–solid process, and hydrothermal method. However, some drawbacks such as the large leakage current usually occurred in the devices prepared by these methods. Through the development of this novel technique, it is expected that the PLD-ZnO nanostructures can be applied for various optoelectronic and microelectronic applications. Additionally, the problem of large leakage current also can be solved efficiently via the fabrication of PLD-ZnO nanostructure-type devices. Most importantly, by choosing suitable seed layers, the novel method will be used for the device fabrications with other oxide materials (such as Ga_2_O_3_, In_2_O_3_, TiO_2_, and so on), expanding the practicality of this growth technique.

## Results and Discussion

**Figure 1:**
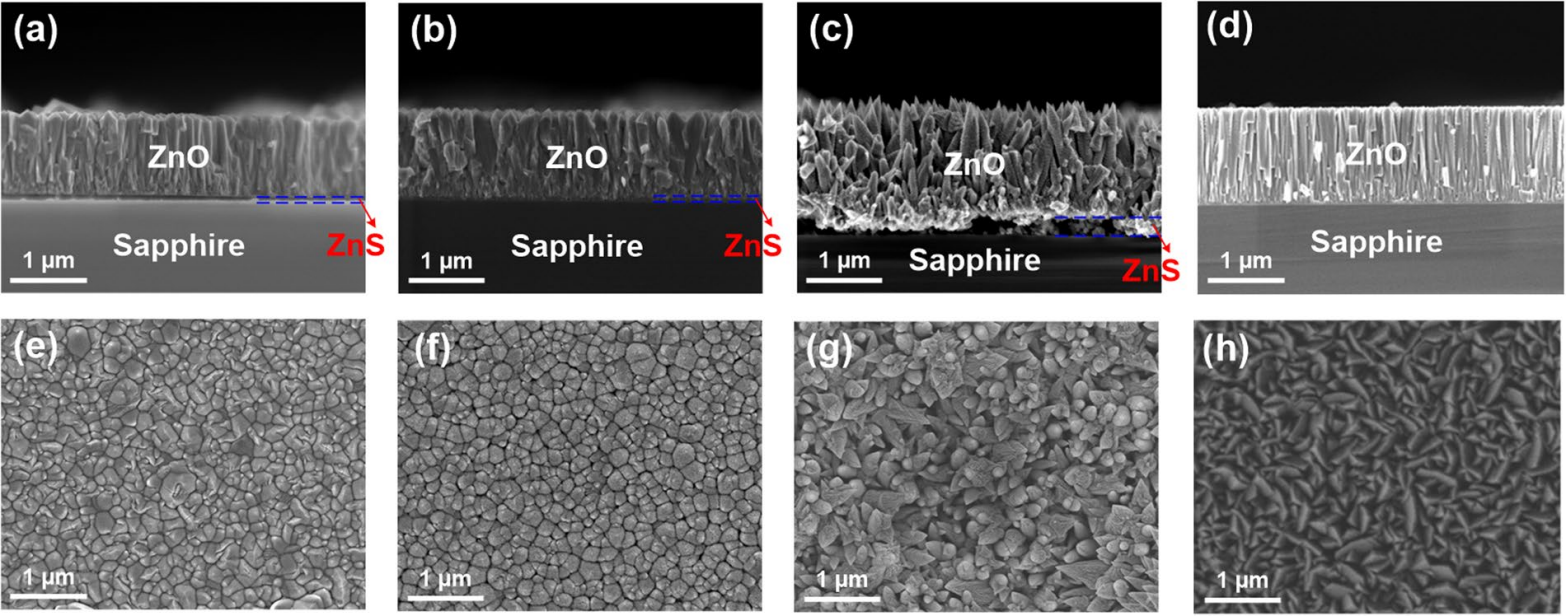
Cross-sectional SEM images of (**a**) 500 °C-grown ZnO/ZnS, (**b**) 700 °C-grown ZnO/ZnS, (**c**) 900 °C-grown ZnO/ZnS, and (**d**) 900 °C-grown ZnO samples. Plan-view SEM images of (**e**) 500 °C-grown ZnO/ZnS, (**f**) 700 °C-grown ZnO/ZnS, (**g**) 900 °C-grown ZnO/ZnS, and (**h**) 900 °C-grown ZnO samples.

The cross-sectional and plan-view SEM images of the PLD-ZnO/CBD-ZnS samples were both taken to observe their morphologies. Figure 1a,b and c shows the cross-sectional SEM images of 500, 700, and 900 °C-grown ZnO/ZnS samples, respectively. Obviously, when the T_s_ was raised to 500 and 700 °C, these two ZnO layers had a similar morphological characteristic to each other. These two ZnO layers both exhibited the columnar structure, and the columns were merged to each other, as shown in Fig. 1a and b. This indicates that the 500 and 700 °C-grown ZnO layers belong to the film-type feature. In addition, it can be found that the thicknesses of these two ZnO films were both approximately 1.1 μm. Further increasing the T_s_ to 900 °C, the morphology of ZnO layer changed to one-dimensional nanostructure-type, as shown in Fig. 1c. We can observe that the 900 °C-grown PLD-ZnO prepared on the CBD-ZnS seed layer was composed of the ZnO nanorods with the diameter approximately 100–200 nm. Besides, the lengths of these nanorods were determined to be 0.7–1.4 μm. The plan-view SEM images of 500, 700, and 900 °C-grown ZnO/ZnS samples are displayed in Fig. 1e,f and g, respectively. Careful observations on Fig. 1e and f revealed that the interval between the columns of the 700 °C-grown ZnO/ZnS was slightly larger than that of the 500 °C-grown ZnO/ZnS. On the other hand, there were countless ZnO nanorods with top tips in the 900 °C-grown ZnO/ZnS, as shown in Fig. 1g. Moreover, Fig. 1d and h displays the cross-sectional and plan-view SEM images of 900 °C-grown ZnO directly prepared on sapphire substrate. Apparently, without growing the CBD-ZnS seed layer, the morphology of PLD-ZnO belonged to film-type even at the T_s_ of 900 °C.

**Figure 2:**
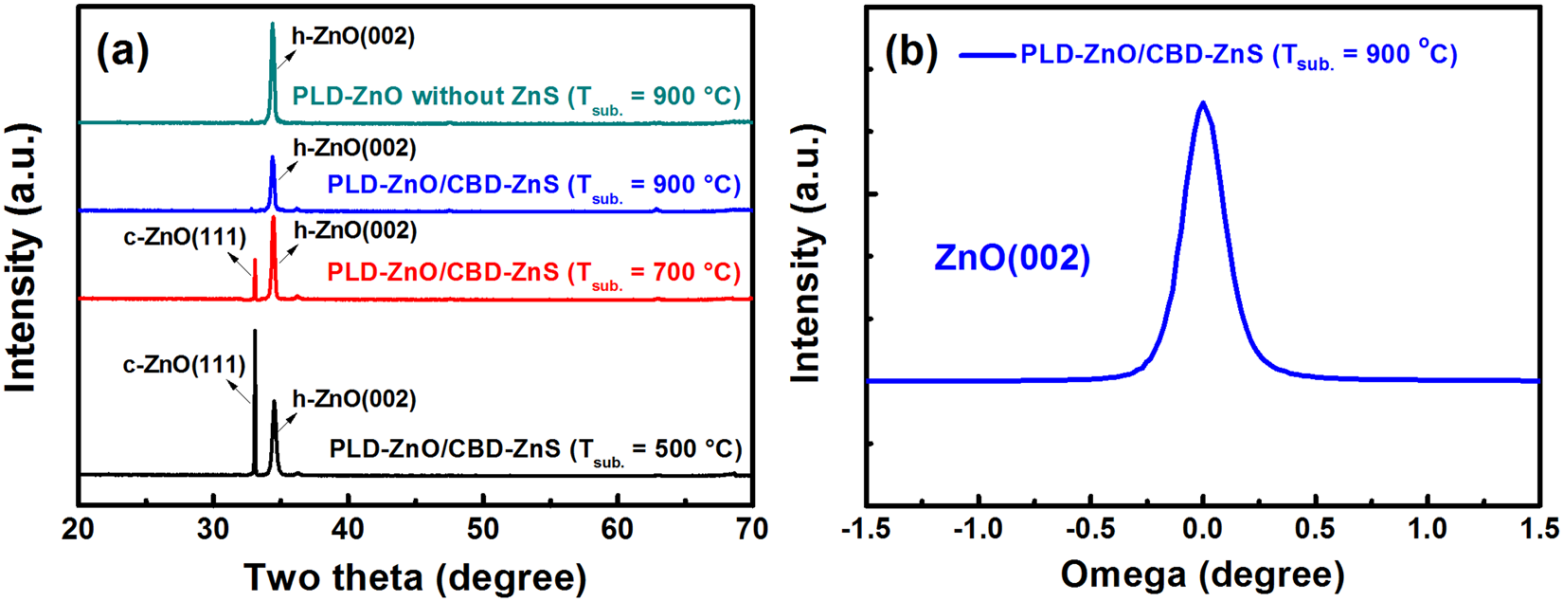
(**a**) XRD patterns of 500–900 °C-grown ZnO/ZnS and 900 °C-grown ZnO (without the ZnS layer) samples. (**b**) XRD rocking curve for the ZnO(002) reflection of the 900 °C-grown ZnO/ZnS sample.

Figure 2a shows the XRD patterns of 500–900 °C-grown ZnO/ZnS and 900 °C-grown ZnO (without the ZnS seed layer) samples. In the XRD patterns of these three ZnO/ZnS samples, there was no diffraction peak indexed to ZnS crystalline phase, indicating that the crystal quality of CBD-ZnS was too low to detect by XRD. When the T_s_ was increased to 500–700 °C, two peaks of cubic ZnO(111) and hexagonal ZnO(002) can be detected in the ZnO/ZnS samples. In general, because of the preferred stacking along (001) plane with the lowest surface free energy on the sapphire surface, ZnO films grown on sapphire substrates always possessed a single crystalline phase with (002) plane family^33^. However, as the PLD-ZnO prepared at T_s_ of 500–700 °C on the CBD-ZnS layer, cubic ZnO(111) and hexagonal ZnO(002) appeared in the meantime. This reveals that the formation of the cubic ZnO(111) phase is mainly attributed to the existence of ZnS seed layer. On the other hand, with increasing the T_s_ from 500 to 700 °C, it can be found that the peak intensity of ZnO(111) reduced significantly. Further increasing the substrate temperature to 900 °C, we can observe that the ZnO/ZnS sample presented a single crystalline structure with the hexagonal ZnO(002) peak. In other words, the cubic ZnO(111) phase disappeared in this sample. In order to investigate the change of the crystalline phase found in the XRD results, we will also perform the TEM observations on these PLD-ZnO/CBD-ZnS samples, as discussed later. Besides, as we mentioned above, only the ZnO(002) diffraction peak existed in the 900 °C-grown ZnO sample. Moreover, the intensity of ZnO(002) diffraction peak in the 900 °C-grown ZnO sample (without the ZnS seed layer) was higher than that of ZnO/ZnS samples. Additionally, to evaluate the crystal quality of ZnO nanorods, the XRD rocking curve measurement was performed, and the full width at half maximum (FWHM) value was analyzed. As shown in Fig. 2b, the XRD FWHM value of the ZnO(002) reflection for the 900 °C-grown ZnO/ZnS sample was determined to be 0.221°. In comparison to the crystal qualities of ZnO nanostructures prepared by conventional methods (such as aqueous solution technique, vapor–liquid–solid process, and hydrothermal method), the crystal quality of ZnO nanorods presented in our work is indeed higher.

**Figure 3:**
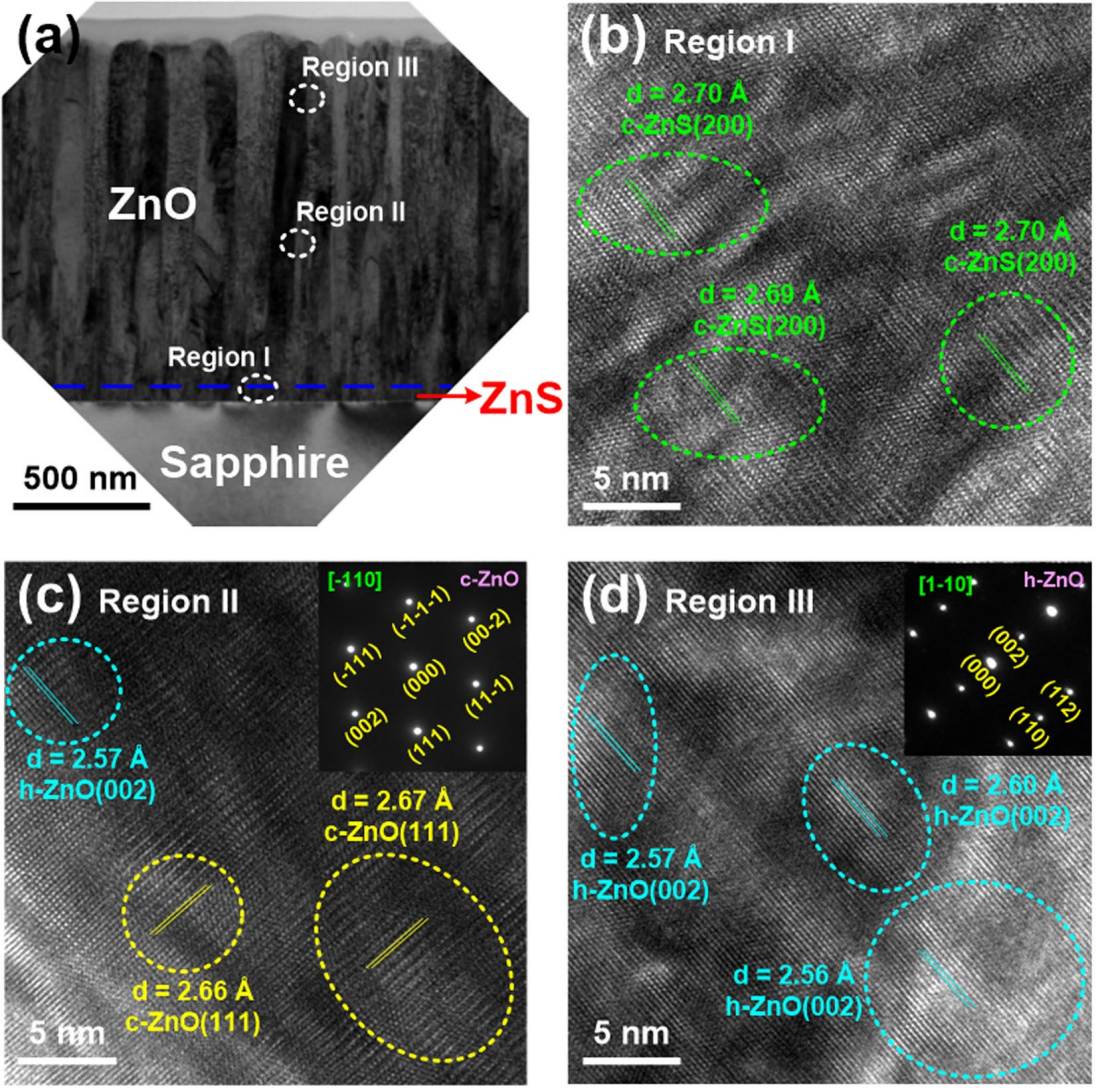
(**a**) Cross-sectional TEM image of 500 °C-grown ZnO/ZnS sample. HR-TEM images focused on the (**b**) region I, (**c**) region II, and (**d**) region III. The area selected electron diffraction patterns for regions II and III are presented, and their zone axes are also marked.

As mentioned above, there is almost no research on the ZnO nanorods grown by conventional PLD on the ZnS seed layer. Thus, we tried to investigate its growth mechanism via the HR-TEM observation. Except for the HR-TEM images taken for these samples, the area selected electron diffraction patterns for ZnO films and nanorods were also analyzed. Figure 3a shows the cross-sectional TEM image of 500 °C-grown ZnO/ZnS sample. It can be seen that the ZnO columns neatly arranged in this image. Then three regions were chosen (marked I, II, and III in Fig. 3a), i.e., the ZnS seed layer, the middle and upper parts of ZnO film to perform the HR-TEM measurements, as exhibited in Fig. 3b,c and d, respectively. Based on our observation in Fig. 3b, the obvious lattice arrangement was found in the ZnS layer, and the d-spacing estimated to 2.69–2.70 Å was indexed to cubic ZnS(200) plane. This implies that the ZnS film belongs to cubic structure after the PLD-ZnO growth at 500 °C. As shown in Fig. 3c, there were two phases of cubic ZnO(111) and hexagonal ZnO(002) appeared in the middle part of ZnO film, where their d-spacings were 2.66–2.67 and 2.57 Å, respectively. In Fig. 3c, the HR-TEM image of region II consisted of cubic and hexagonal ZnO phases. However, the area selected electron diffraction pattern of region II belonged to cubic ZnO phase. This could be attributed that the ratio of hexagonal ZnO phase in region II is very low. In the upper part of ZnO film (Fig. 3d), the crystalline phase was identified to hexagonal ZnO(002) with the d-spacing of 2.56–2.60 Å.

**Figure 4:**
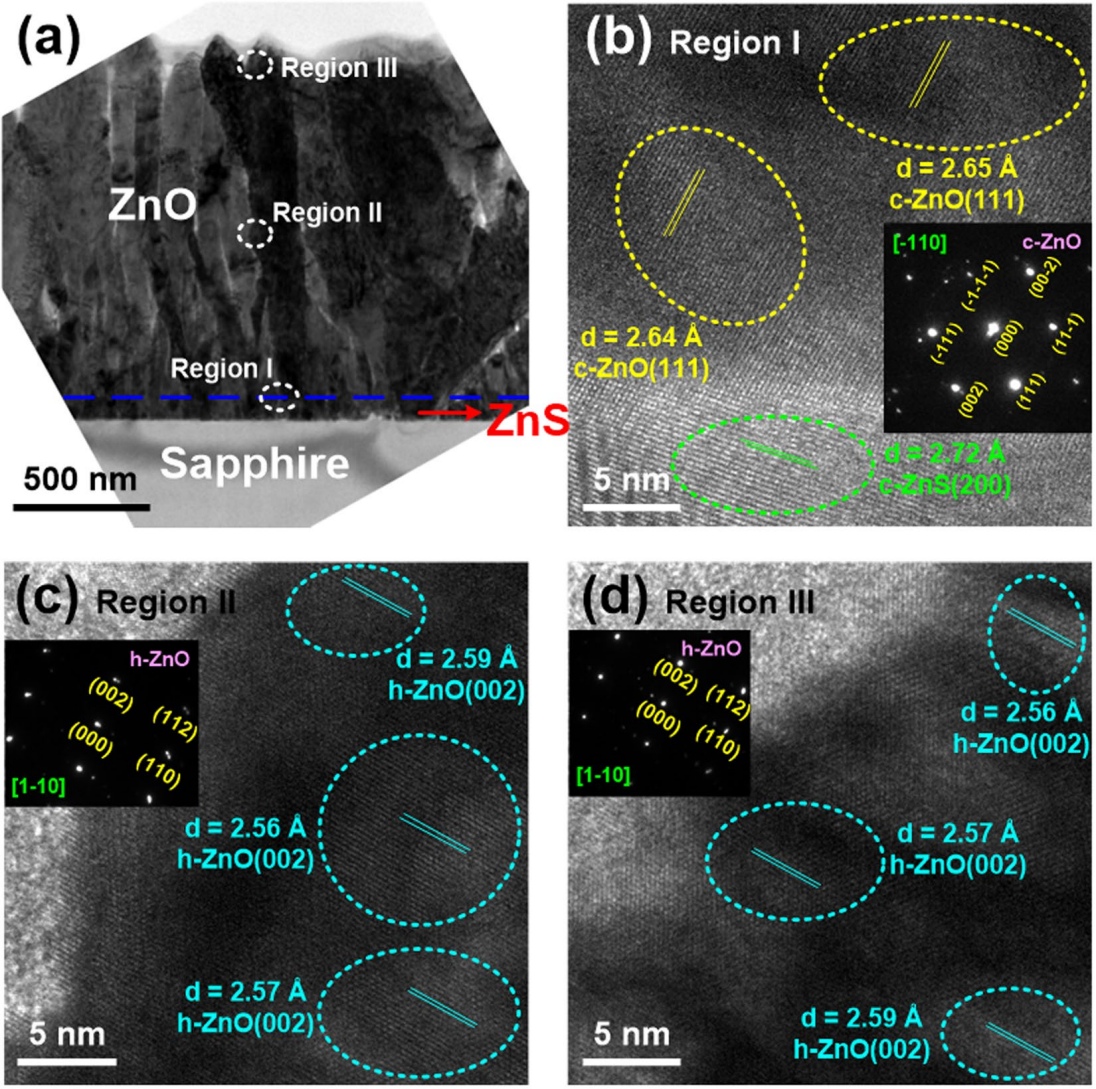
(**a**) Cross-sectional TEM image of 700 °C-grown ZnO/ZnS sample. HR-TEM images focused on the (**b**) region I, (**c**) region II, and (**d**) region III. The area selected electron diffraction patterns for regions I, II, and III are presented, and their zone axes are also marked.

The cross-sectional TEM image of 700 °C-grown ZnO/ZnS sample is displayed in Fig. 4a. We also selected three regions (marked I, II, and III in Fig. 4a) to observe their HR-TEM images, as shown in Fig. 4b,c and d, respectively. These three regions indicated the interface between ZnS and ZnO, the middle and upper parts of ZnO film, respectively. In comparison to the 500 °C-grown ZnO/ZnS sample, similar TEM results were observed in this sample. In Fig. 4b, the d-spacing of ZnS layer was 2.72 Å, confirming that the ZnS film can be classified as the cubic ZnS(200) phase. Moreover, in region I, the ZnO film possessed the cubic ZnO(111) phase with the d-spacing of 2.64–2.65 Å. According to the analyses in regions II and III, the d-spacings were evaluated to 2.56–2.59 Å, which revealed that the crystalline structures of these two regions were both hexagonal ZnO(002) phase.

**Figure 5:**
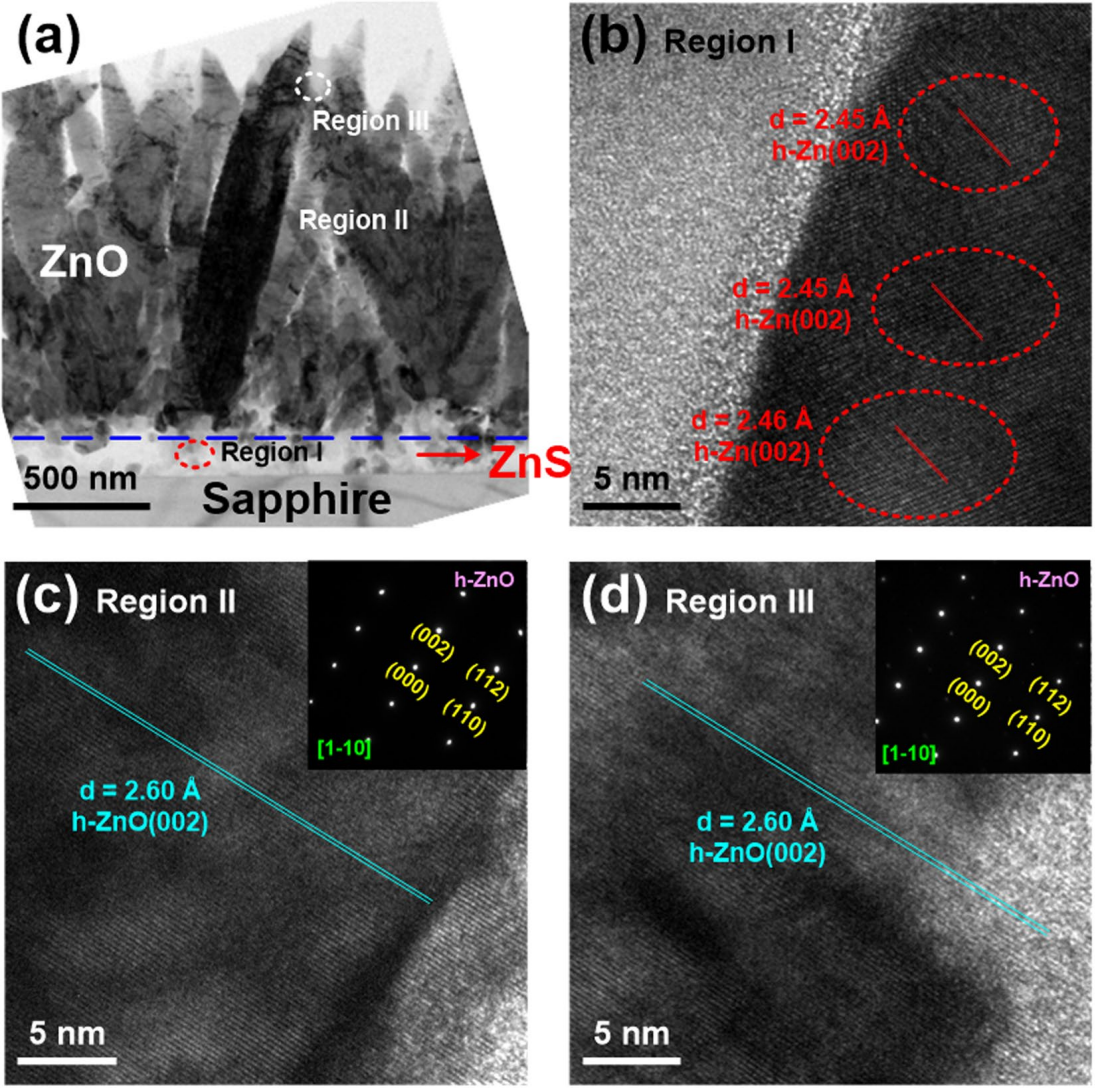
(**a**) Cross-sectional TEM image of 900 °C-grown ZnO/ZnS sample. HR-TEM images focused on the (**b**) region I, (**c**) region II, and (**d**) region III. The area selected electron diffraction patterns for regions II and III are presented, and their zone axes are also marked.

The microstructures of the 900 °C-grown ZnO/ZnS sample were also investigated by TEM, as shown in Fig. 5. The cross-sectional TEM image of this sample is presented in Fig. 5a. Three regions (I, II, and III) in Fig. 5a were also taken by HR-TEM to realize the growth mechanism of ZnO nanorods. In region I (Fig. 5b), the lattice of this region was indexed to hexagonal Zn(002) phase with the d-spacing of 2.45–2.46 Å, and there was no existence of ZnS phase. It was apparent that the crystal structure of ZnS film was transformed from cubic ZnS to hexagonal Zn after depositing the ZnO layer at the T_s_ of 900 °C. Then we observed the HR-TEM images of regions II and III, the d-spacings for middle and upper parts of ZnO nanorods were both 2.60 Å, revealing ZnO nanorods possessed hexagonal ZnO(002) phase. By combining XRD and TEM results, the single crystalline hexagonal ZnO(002) phase presented in the 900 °C-grown ZnO/ZnS sample was easily formed on the hexagonal Zn(002) phase. Very interestingly, with increasing the T_s_ to 900 °C, the ZnS film became a sacrifical layer and the Zn structure was formed on sapphire substrate. Additionally, the formation of the hexagonal Zn phase was helpful to the preparation of one-dimensional ZnO nanostructure.

**Figure 6:**
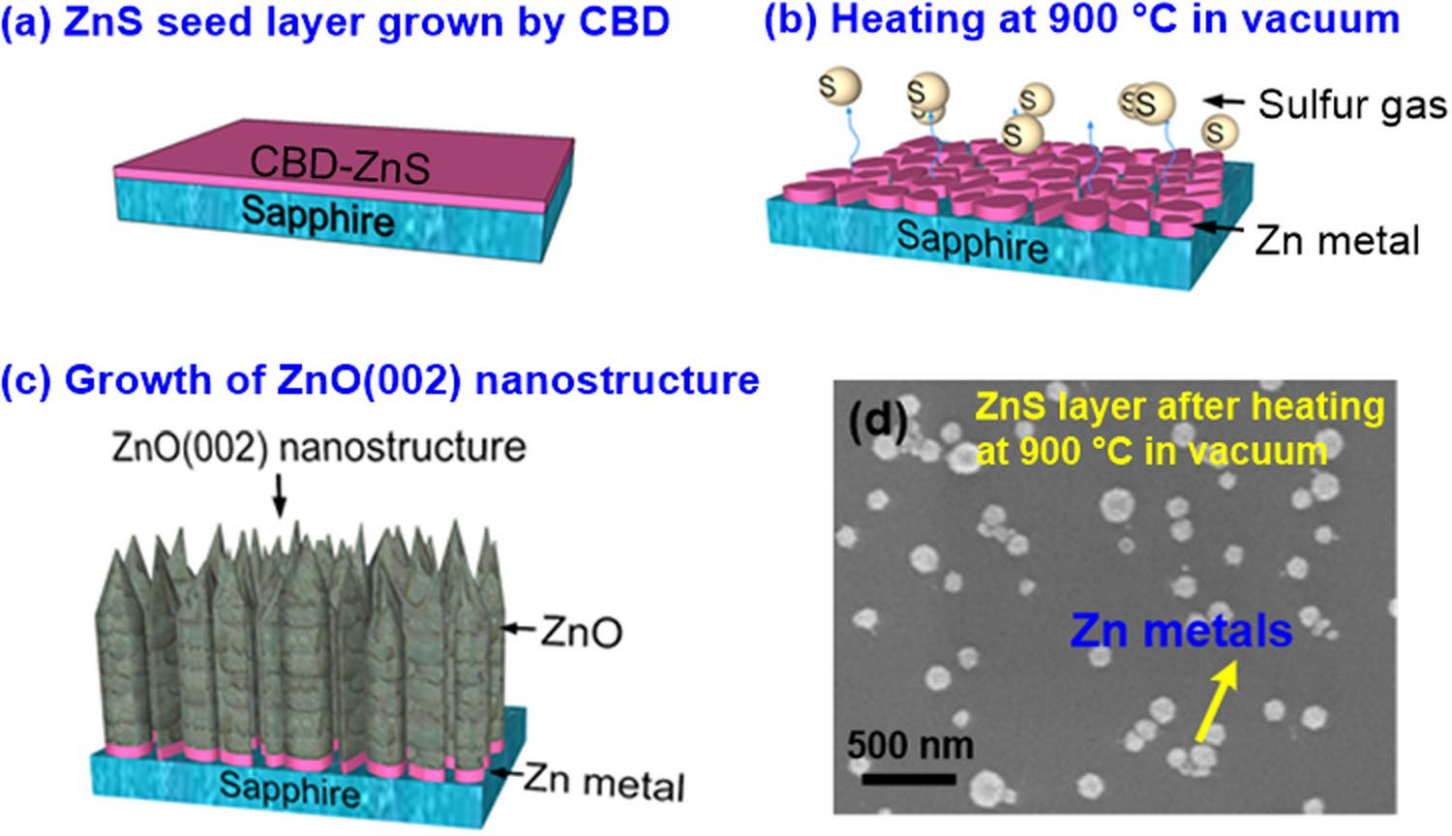
Growth mechanism of 900 °C-grown PLD-ZnO on the CBD-ZnS seed layer: (**a**) ZnS growth by CBD, (**b**) heating sample at 900 °C in vacuum, and (**c**) growth of hexagonal ZnO(002) nanostructure by PLD. (**d**) Plan-view SEM image of the ZnS seed layer after heating at 900 °C for 5 min in the vacuum environment.

Figure 6 shows the growth mechanism of single crystalline ZnO(002) nanorods by PLD at 900 °C on the ZnS seed layer. In Fig. 6a, the ZnS seed layer was prepared on sapphire via the CBD method. At this state, the ZnS belonged to film-type morphology. The ZnS/sapphire was then put into the PLD chamber at a vacuum atmosphere, and the sample was heated from room temperature to 900 °C (Fig. 6b). Based on the TEM observations of 500 and 700 °C-grown ZnO/ZnS samples (Figs 3 and 4), the crystal structure of ZnS seed layer was indexed to cubic ZnS. Moreover, it could be speculated that the ZnS seed layer still belonged to film-type structure after heating at 500 and 700 °C. However, when the PLD-ZnO was prepared at 900 °C, the crystal structure of ZnS seed layer was transformed from cubic ZnS to hexagonal Zn. Commonly, as the temperature was higher than 900 °C, the ZnS can be decomposed with the release of zinc metals and sulfur fumes. In other words, after the thermal decomposition of ZnS ⇔ Zn(s) + S(g), the sulfur fumes were exhausted and the Zn metals were formed on sapphire. The Zn metals have a hexagonal crystal structure. Because of the preferred stacking along (002) plane with the lowest surface free energy on the substrate surface, the Zn grains were formed with the (002) orienation, leading to the subsequent growth of single crystalline ZnO, as shown in Fig. 5. Additionally, we can deduce that many small Zn grains were merged together to larger grains during the high-temperature process. Finally, the PLD-ZnO nanostructure with a single crystalline hexagonal ZnO(002) phase was grown at 900 °C, as shown in Fig. 6c. Obviously, with increasing the substrate temperature to 900 °C, the microstructure transformation of ZnO layer was attributed to the change of seed layer from the ZnS film to the Zn grains. Figure 6d shows the plan-view SEM image of the ZnS seed layer after heating at 900 °C for 5 min in the vacuum environment. After heating at 900 °C, the ZnS layer doesn’t show the film-type morphology. In the SEM image, we can observe that several islands formed on sapphire substrate, and these islands were identified to Zn metals by energy dispersive spectroscopy. The result is in good agreement with our deduction, as shown in Fig. 6b. In addition, the change from the ZnS film to a sacrifical layer after heating the substrate at 900 °C also can be clearly observed in Fig. 6d. Currently, few researches related to the ZnO nanostructures/Zn layers have been presented, where Zn layers and ZnO nanostructures were grown by evaporation and hydrothermal method, respectively^34,35^. Since evaporation and hydrothermal methods are both the low-temperature processes, these ZnO nanostructures deposited on the evaporated Zn layers are polycrystalline with low crystal qualities. It should be noted again that the single crystalline ZnO nanorods prepared on the ZnS seed layer can be achieved in our novel fabrication method.

**Figure 7:**
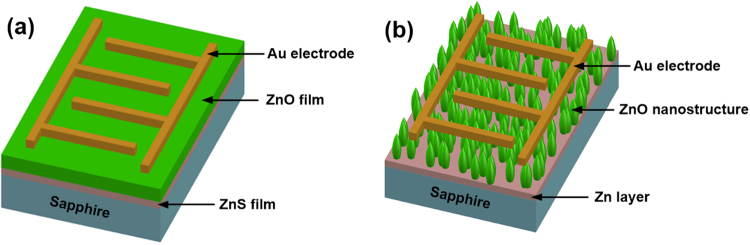
Schematic diagrams of MSM PDs with (**a**) film-type and (**b**) nanostructure-type ZnO layers.

**Figure 8:**
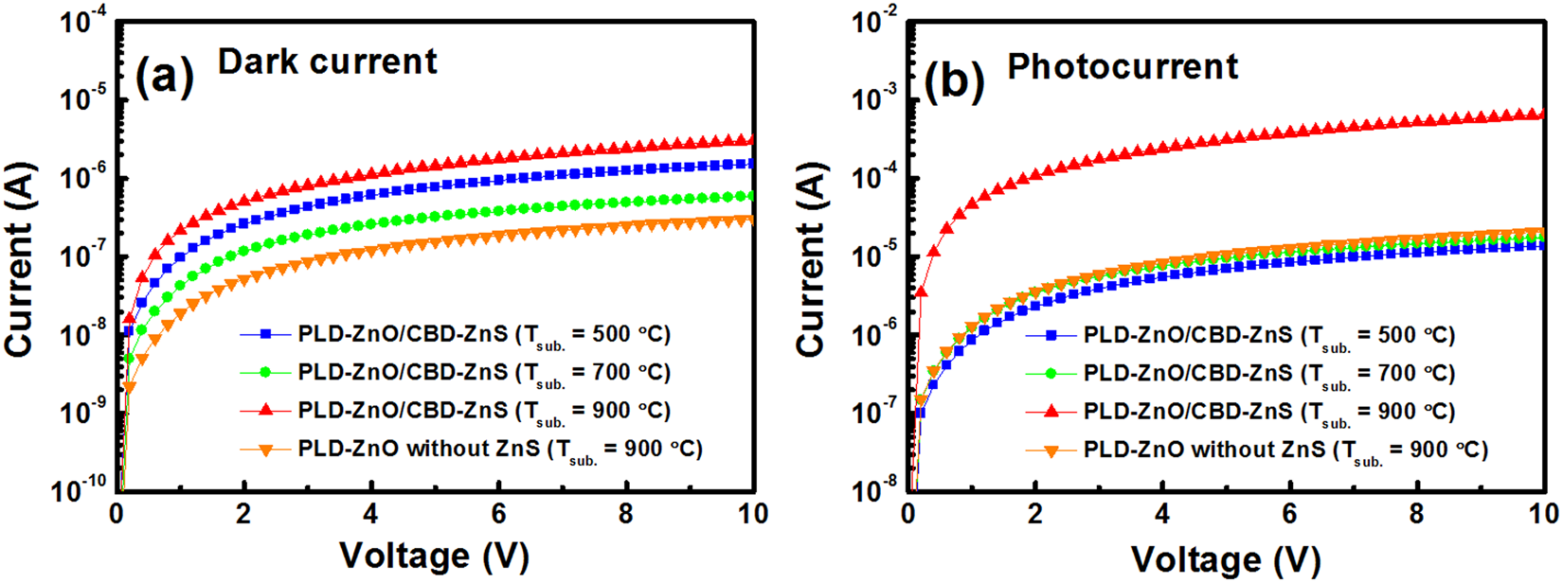
Room temperature *I*–*V* characteristics of MSM PDs with 500, 700, 900 °C-grown ZnO/ZnS and 900 °C-grown ZnO samples measured (**a**) in the dark and (**b**) under 370 nm illumination.

Then, the 500–900 °C-grown ZnO/ZnS and 900 °C-grown ZnO (without the ZnS seed layer) samples were all selected to fabricate the MSM PDs and investigated their device performances. Figure 7a and b shows the schematic diagrams of these devices with the film-type and nanostructure-type ZnO layers, respectively. As mentioned above (Fig. 1), the PDs fabricated with 500–700 °C-grown ZnO/ZnS and 900 °C-grown ZnO samples belong to film-type devices. Meanwhile, the PD prepared with the 900 °C-grown ZnO/ZnS is nanostructure-type device. The size of interdigitated pattern on the shadow mask was 2000 μm wide and 2200 μm long. Moreover, the finger width and interspacing were both 100 μm. Figure 8a and b presents the room temperature *I*–*V* characteristics of these MSM PDs measured in the dark (for dark current) and under 370 nm illumination (for photocurrent). As shown in Fig. 8a, it can be seen that the dark current of the PD prepared with the 900 °C-grown ZnO/ZnS was slightly larger than that with the other three samples. This implies that the dark current (under forward voltage) of ZnO-nanostructure PD is a little larger than that of ZnO-film PD. Under an applied bias of 1 V, the dark currents of the PDs with the 500, 700, 900 °C-grown ZnO/ZnS and 900 °C-grown ZnO were measured to be 9.62 × 10^−8^, 4.23 × 10^−8^, 2.15 × 10^−7^, and 1.92 × 10^−8^ A, respectively. In addition, under UV illumination (370 nm), the photocurrents (@1 V) of these four devices were analyzed to be 8.65 × 10^−7^, 1.26 × 10^−6^, 4.62 × 10^−5^, and 1.32 × 10^−6^ A, respectively, as shown in Fig. 8b. For the PD device, the signal-to-noise ratio is defined as the photocurrent to dark current contrast ratio. Therefore, after our calculations, the signal-to-noise ratios (@1 V) of these four PDs were 9.0, 29.8, 214.9, and 68.8, respectively. Obviously, both the larger photocurrent and signal-to-noise ratio can be obtained in the ZnO-nanostructure PD. Because the ZnO nanostructure has a larger surface area in comparison to that of the ZnO film, resulting in an increment of light trapping to facilitate oxygen adsorption and desorption at the ZnO nanorod surface. The definition of increased light trapping is the further light absorption by increasing the optical path length in the active layer. This is the reason why the ZnO-nanostructure PD can possess a better signal-to-noise characteristic.

**Figure 9:**
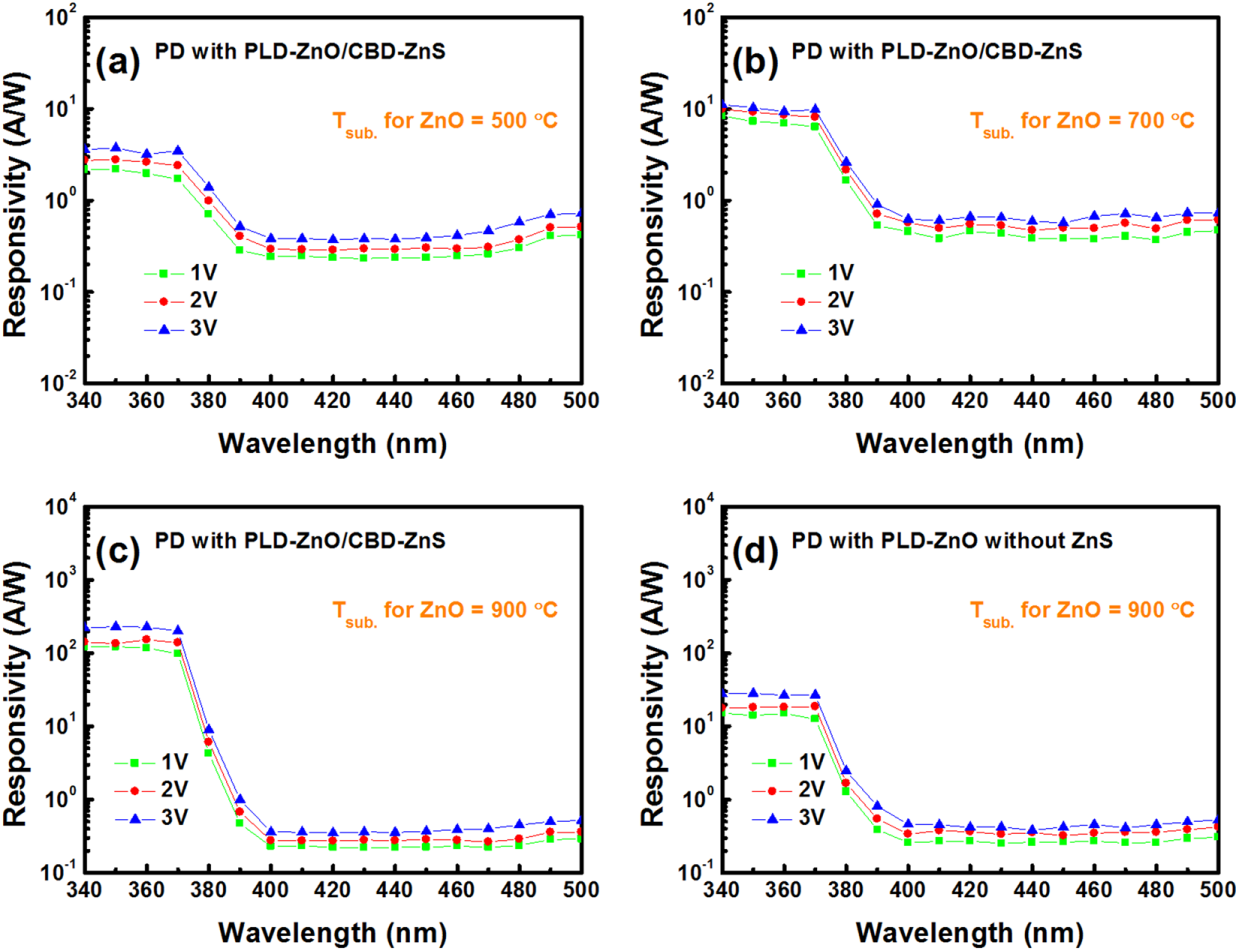
Responsivities as a function of wavelength for the MSM PDs prepared with (**a**) 500 °C-grown ZnO/ZnS, (**b**) 700 °C-grown ZnO/ZnS, (**c**) 900 °C-grown ZnO/ZnS, and (**d**) 900 °C-grown ZnO samples.

The responsivities as a function of wavelength for the MSM PDs prepared with the 500, 700, 900 °C-grown ZnO/ZnS and 900 °C-grown ZnO samples are displayed in Fig. 9a,b,c and d, respectively. Here, the bias voltages of 1, 2, and 3 V were used in the measurements. The wavelength was raised from 340 to 500 nm during these measurements. We can observe that the cutoff clearly occurred at around 370 nm for these four devices, corresponding to the bandgap energy of ZnO (3.37 eV). Besides, the PD prepared with the 900 °C-grown ZnO/ZnS sample (nanostructure-type) had a more obvious cut-off feature than the other three devices (film-type). On the other hand, as the bias voltage was increased, these three PDs all presented the increasing responsivity. This demonstrates that the devices have a large photoconductive gain^36^. When a bias voltage of 1 V was applied, the responsivities (@370 nm) of these MSM PDs with the 500, 700, 900 °C-grown ZnO/ZnS and 900 °C-grown ZnO samples were 1.71, 6.35, 98.67, and 12.66 A/W, respectively. Meanwhile, the responsivities (@450 nm) of these four devices were 0.237, 0.3856, 0.2247, and 0.263 A/W, respectively. Therefore, for the measured wavelength range between 370 and 450 nm, the contrast ratios (UV-to-visible discrimination ratios) of these four MSM PDs were determined to be 7.2, 16.5, 439.1, and 48.1, respectively. Among these three film-type devices, the optoelectronic performances of the PD fabricated with the 900 °C-grown ZnO are better than those with the 500 and 700 °C-grown ZnO/ZnS samples. This could be attributed that the 900 °C-grown ZnO film possesses a higher crystal quality than the other two films. Besides, the responsive performance of the PD with the 900 °C-grown ZnO/ZnS sample is about one order higher than that with the 900 °C-grown ZnO film. Due to the growth of one-dimensional nanostructure in the 900 °C-grown ZnO/ZnS sample, a higher light-capturing efficiency can be obtained. In addition, the single crystalline structure formed in the sample is also helpful to improve the device performance^24^.

Table 1 presents a summary of the solar-blind MSM PDs fabricated with ZnO-based films and nanostructures by various techniques. At present, most of film-type ZnO-based PDs were prepared by sputtering, and these ZnO-based films almost exhibited single crystalline structure with a preferred growth orientation of (002) plane^7–9^. Although the single crystalline structure can be easily achieved in the sputtered ZnO films, the relatively smaller surface area of the film-type PDs (in comparison to that of the nanostructure-type PDs) would degrade their device performance. To improve the performance of the film-type PDs, the enhancement in the crystal quality of ZnO film via the post-annealing process is an efficient method^9^. On the other hand, the nanostructure-type ZnO-based PDs were almost fabricated by aqueous solution^10,13,15^ and hydrothermal growth^16–19^ methods. Due to the low-temperature processes of aqueous solution and hydrothermal growth methods, these ZnO-based nanostructures all possessed the polycrystalline structures with low crystal qualities. Some methods were used for enhancing the performance of nanostructure-type ZnO-based PDs. For example, via the fabrication of the laterally aligned ZnO nanorods, the device performance can be improved efficiently, which could be resulted from the significant increment of the surface area^16^. Additionally, by doping various elements into ZnO, the performance of nanostructure-type PDs can be also enhanced^13,18,19^. In this study, the MSM PD with the single crystalline ZnO(002) nanorods can be fabricated by PLD at the T_s_ of 900 °C. This device has both a high crystal quality and the large surface area, leading to its high optoelectronic performance. In the future, we will prepare the PDs with ZnO-based nanostructures doped with various elements to further improve their optoelectronic performance. Actually, a variety of materials can be prepared by CBD method. If suitable seed layers are prepared by CBD, various oxides nanostructures can be grown by the novel technique presented in this study. Except for the better optoelectronic performance, a higher electronic stability (a smaller leakage current) could be obtained in the nanostructure-type devices fabricated using this novel technique in comparison to that by aqueous solution technique, vapor–liquid–solid process, and hydrothermal method.

**Table 1:** Summary of the solar-blind MSM PDs fabricated with ZnO-based films and nanostructures by various techniques.

Growth method	Structure	device performance	Ref.
Sputtering	Single crystalline ZnO film	Max. responsivity (@100 V): 0.35 A/W UV-to-visible discrimination ratio (@100 V): > 300	7
Sputtering	Single crystalline ZnO film	Max. responsivity (@5 V): 3.6 × 10^−4^ A/W	8
Sputtering & post-annealing	Single crystalline Ga-doped ZnO film	Max. responsivity (@10 V): 54 A/W	9
Aqueous solution	Polycrystalline In-doped ZnO nanosheets	Max. responsivity (@1 V): 0.074 A/W UV-to-visible discrimination ratio (@1 V): 312	10
Aqueous solution	Polycrystalline Ga-doped ZnO nanosheets	Max. responsivity (@1 V): 5.75 A/W UV-to-visible discrimination ratio (@1 V): 36.1	13
Aqueous solution	Polycrystalline ZnO nanosheets	Max. responsivity (@1 V): 0.204 A/W UV-to-visible discrimination ratio (@1 V): 42	15
Hydrothermal growth	Laterally aligned ZnO nanorods	Max. responsivity (@1 V): 120 A/W	16
Hydrothermal growth	Polycrystalline ZnO nanorods	Max. responsivity (@0.5 V): 22.1 A/W	17
Hydrothermal growth	Polycrystalline Co-doped ZnO nanorods	Max. responsivity (@1 V): 19.8 A/W	18
Hydrothermal growth	Polycrystalline Fe-doped ZnO nanorods	Max. responsivity (@1 V): 0.06 A/W UV-to-visible discrimination ratio (@1 V): 23	19
PLD	Single crystalline ZnO nanorods	Max. responsivity (@1 V): 98.67 A/W UV-to-visible discrimination ratio (@1 V): 439.1	This study

The structures of ZnO-based materials and device performances of PDs are compared.

## Conclusion

We have presented a novel technique for the growth of single crystalline ZnO nanorods by conventional PLD combined with a sacrifical nanostructure. The PLD-ZnO layers were prepared at 500–900 °C on the CBD-ZnS seed layers. As the T_s_ was heated to 500–700 °C, the ZnO grown on the cubic ZnS layer belonged to the film-type morphology and polycrystalline structure. However, with increasing the T_s_ to 900 °C, the ZnS seed layer became a sacrifical layer and the hexagonal Zn(002) structure was formed through the thermal decomposition of ZnS. This leads to the formation of single crystalline ZnO(002) nanorods in the 900 °C-grown ZnO/ZnS sample. After fabricating the MSM PDs with the 500, 700, 900 °C-grown ZnO/ZnS and 900 °C-grown ZnO samples, the dark currents (@1 V) of these four devices were 9.62 × 10^−8^, 4.23 × 10^−8^, 2.15 × 10^−7^, and 1.92 × 10^−8^ A, while their photocurrents (@1 V) were 8.65 × 10^−7^, 1.26 × 10^−6^, 4.62 × 10^−5^, and 1.32 × 10^−6^ A, respectively. Obviously, the PD fabricated with the 900 °C-grown ZnO/ZnS sample (nanostructure-type) has a much higher signal-to-noise ratio of 214.9 than the other three devices (film-type). Additionally, this device also possesses an apparently better responsive performance, where its responsivity (@1 V and 370 nm) and UV-to-visible discrimination ratio were 98.67 A/W and 439.1, respectively, which are about one order higher than those with the 900 °C-grown ZnO film. The significant improvement in the optoelectronic performance of the PD with the 900 °C-grown ZnO/ZnS sample can be attributed to both the formation of one-dimensional nanostructure and its high crystal quality. The results reveal that the novel growth technique using PLD with the sacrifical nanostructure is highly potential for high performance ZnO-based devices.

## Methods

In this study, the ZnO layers were deposited on ZnS seed films to fabricate the PDs. Firstly, ZnS thin films with a thickness approximately 80 nm were grown on c-plane sapphire substrates using the CBD method^37^. The zinc sulfate (ZnSO_4_) and thiourea (SC(NH_2_)_2_) solutions were employed in the CBD growth, and their concentrations were kept at 1.4 × 10^−3^ and 0.105 M, respectively. To prepare a stable complex with zinc ions, ZnSO_4_ and 28%-30% NH_4_OH solutions were mixed in a glass beaker and then stirred for 10 min. Similarly, in another glass beaker, SC(NH_2_)_2_ was added into the 98 + % hydrazine hydrate solution with constant stirring for 10 min. Subsequently, these two solutions were mixed with each other in a glass tank that placed on a hotplate-stirrer. Finally, the substrate was soaked in this mixed solution at a reaction temperature of 85 °C. After immersing the substrate for 2 hours, the growth of ZnS seed layer was completed and the sample can be taken out.

Next, ZnO films were prepared on ZnS seed layers by conventional PLD (PLD/MBE-2000, PVD products) with a KrF excimer laser source (λ = 248 nm). A stoichiometric ceramic ZnO target with a diameter of 3 inch was ablated via the laser radiation. The repetition rate, energy fluence, and duration of the pulsed laser were fixed at 1 Hz, 600 mJ/pulse, and 25 ns, respectively. During the ZnO growth, the T_s_ can be increased to 500, 700, and 900 °C using a resistive heater. The ZnO layer was deposited with 12000 laser pulses. Moreover, the distance between target and substrate was 8 cm. As the base pressure was lower than 1 × 10^−7^ Torr, the O_2_ gas was introduced into the deposition chamber, and the working pressure was maintained at 1 × 10^−2^ Torr.

Crystal structure of the PLD-ZnO/CBD-ZnS sample was analyzed by x-ray diffraction (XRD) (PANalytical, X’Pert Pro MRD). In the XRD equipment, Cu K_α_ radiation and Ge (220) were served as the source and the monochromator, respectively. Surface morphology and thickness of the sample were investigated by field-emission scanning electron microscopy (FE-SEM). To realize the growth mechanism of the PLD-ZnO prepared on the CBD-ZnS, high-resolution transmission electron microscopy (HR-TEM, model: JEM-2100 F) was used to observe the sample’s microstructures.

The PLD-ZnO layers grown at 500, 700, and 900 °C on the CBD-ZnS were all employed to fabricate the MSM PDs. To form the Schottky contact metal, the 100-nm-thick Au film was deposited on the sample by thermal evaporation. The interdigitated contact electrodes were fabricated through standard photolithography and wet etching processes in sequence. Then, a HP 4156 semiconductor analyzer was used to characterize the current–voltage (I-V) properties of these MSM PDs at room temperature. Through the measurements using a Jobin-Yvon Spex system with a 300 W xenon arc lamp light source and the standard synchronous detection scheme, the spectral responsivities of these devices can be obtained. In addition to the PDs fabricated with the PLD-ZnO/CBD-ZnS samples, we also prepared the device with the ZnO layer directly grown on sapphire (T_s_: 900 °C) as a contrasted sample.
